# The Role of the Gut Microbiome in Inflammatory Bowel Disease: The Middle East Perspective

**DOI:** 10.3390/jpm14060652

**Published:** 2024-06-18

**Authors:** Ahmed El-Sayed, Diya Kapila, Rama Sami Issa Taha, Sherif El-Sayed, Mohd Rafiw Ahmed Mahen, Roa’a Taha, Laith Alrubaiy

**Affiliations:** 1 Hillingdon Hospital NHS Trust, London UB8 3NN, UK; ahmed.el-sayed@nhs.net (A.E.-S.); d.kapila@nhs.net (D.K.); 2 Healthpoint Hospital, Abu Dhabi P.O. Box 112308, United Arab Emirates; rama.sami.issa.taha060@med.tsu.edu.ge (R.S.I.T.); roaa.sami.issa.taha450@med.tsu.edu.ge (R.T.); 3 Mid and South Essex NHS Trust, London SS0 0RY, UK; sherif.el-sayed3@nhs.net; 4 Department of Medicine, King’s College Hospital London, Dubai P.O. Box 340901, United Arab Emirates; rafiw.mahen@kch.ae; 5 College of Medicine and Health Sciences, Khalifa University, Abu Dhabi P.O. Box 127788, United Arab Emirates

**Keywords:** gut microbiome, inflammatory bowel disease, ulcerative colitis, Crohn’s disease, Middle East

## Abstract

The gut microbiome is of paramount importance in preserving internal balance in the gastrointestinal tract; therefore, disruptions in its regulation have been linked to the development of inflammatory bowel disease (IBD). This article explores the intricate details of the gastrointestinal microbiome as it pertains to inflammatory bowel disease (IBD), with an emphasis on the Middle East. The study reviews the typical gut microbiome, modifications in inflammatory bowel disease (IBD), determinants impacting the gut microbiome of the Middle East, and prospective therapeutic interventions.

## 1. Introduction

### 1.1. Background

Ulcerative colitis (UC) and Crohn’s disease (CD) are the two leading forms of inflammatory bowel disease (IBD), a condition that is affected by both genetic and environmental factors [1]. Although it is widespread globally, its prevalence is highest in western nations; however, there are also indications of an upward trend in the Arab world [2]. IBD cases are projected to have increased substantially by 2035 [3], necessitating an associated rise in resources.

Understanding the interplay between disease susceptibility and the dynamics of the human microbiome is important and may potentially allow us to develop personalised therapies in future [4]. The microbiome is composed of an incredible array of bacteria with a wide variety of functions, from *Firmicutes* and the conversion of fibre into short chain fatty acids, vital for multiple important functions [5] to *Bacteroidetes* and their role in signalling colonic immune cells [6].

Patients with IBD have been noted to have differences in their microbiome composition compared to the general population, such as an increase in *Protobacteria* and *E. Coli* and a decrease in *Firmicutes* and *Eubacterium* [7]. Indeed, further analysis has shown that a reduction in *F. prausnitzii* is often found in patients with Crohn’s disease and seems to be predictive of more severe disease [8].

Despite this, changes are not uniform. As the Middle East has industrialised, its diet has changed, along with an increased incidence of IBD [2]. Moreover, genetic markers, which seem to be prevalent indicators in Caucasian populations, have been found to not necessarily correlate with Middle Eastern populations [9].

As our understanding evolves further, probiotics, targeted dietary changes, and even faecal transplants may all provide options for precise, personalised microbiome modulation as a therapeutic option in future.

### 1.2. Objectives

The article aims to highlight the bacteria that make up the normal microbiome, as well as their normal functions and factors that affect them, both globally and within the Middle East. There is also a focus on outlining the role of the microbiome in IBD, regional variations, and how this knowledge may be used to develop more personalised interventions in future.

### 1.3. Methods

We conducted a comprehensive literature review utilising PubMed, MEDLINE, Embase, and Google Scholar to identify relevant journal articles. The following keywords were used (Table 1). Studies on both adults and children were included where appropriate.

## 2. Normal Gut Microbiome

### 2.1. Composition

The bacteria involved in our microbiota differ between areas and are thought to be important for our health [4,10]. They perform several tasks, including vitamin synthesis, food fermentation, pathogen defence, and immune stimulation. Six phyla predominantly make up the gut microbiota: *Firmicutes*, *Bacteroidetes*, *Actinobacteria*, *Proteobacteria*, *Fusobacteria*, and *Verrucomicrobia* [11], with *Firmicutes* and *Bacteroidetes* being predominant [5].

*Firmicutes* help to break down many substrates, generating metabolites [5]. The genera within, such as *Lactobacillus*, *Clostridium*, and *Bacillus*, have distinct roles in the gastrointestinal tract. Overall, they help convert dietary fibre into butyrate, a chemical created during microbial fermentation, required for colonic epithelial maintenance, histone deacetylation, and gene expression, and which has been demonstrated to lower the risk of IBD and colorectal cancer [12,13].

*Firmicutes* also ferment complex carbohydrates and dietary fibres to produce short-chain fatty acids (SCFAs), including butyrate, propionate, and acetate [14]. SCFAs have anti-inflammatory properties, provide colonic epithelial cells with energy, and support the integrity of the intestinal barrier [14]. Additionally, several *Firmicutes* species have been linked to the synthesis of antimicrobial peptides, which support a healthy gut microbial ecosystem and help defend against pathogens [15].

The *Bacteroidetes* species, including *Prevotella* and *Bacteroides*, is known for its capacity to break down polysaccharides and alter immunological reactions [16]. *Bacteroides’* capacity to use polysaccharides as a source of nutrients may also help support the symbiotic interaction between gut bacteria and others. The *Bacteroides* species possess polysaccharide utilisation loci, gene clusters that encode systems for breaking down polysaccharides [17], which are subsequently fermented by gut bacteria into SCFAs, hydrogen, carbon dioxide, and other metabolites that are directly linked to the health of the host [18].

Colonic regulatory T cells (Tregs) identify colonic contents, and *Bacteroides fragilis* can signal Tregs to reduce pro-inflammatory T-helper 17 (Th17) responses [6]. The microbiota also play a crucial role in the activity of intestinal CD8+ T cells, influencing peripheral immune cells such as natural killer cells, plasmacytoid DCs, and marginal zone B cells. Disruptions in the gut microbiota may therefore result in a more pro-inflammatory state [19].

By stimulating the development of Tregs and preserving a careful balance between pro- and anti-inflammatory signals, bacteria play a critical role in developing immunological tolerance. *Bacteroidetes* inhibit aberrant immune activation and the emergence of inflammatory illnesses like IBD and allergies by interacting with the host immune system [6]. *Firmicutes* and *Bacteroidetes* are therefore essential for reducing inflammation [6,15].

**Figure 1:**
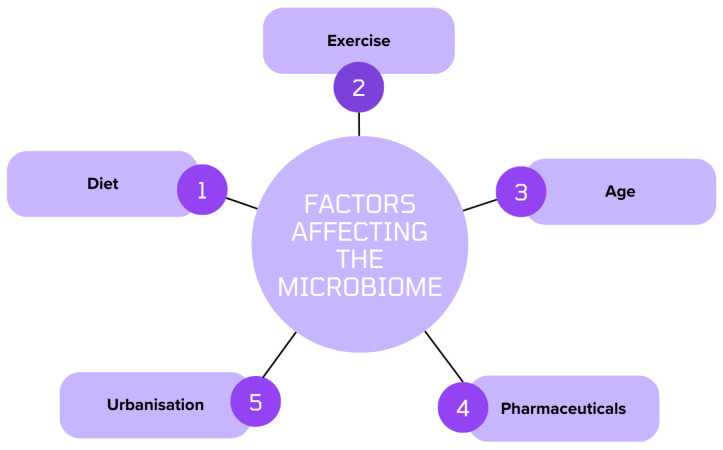
Factors Affecting the Microbiome.

The microbiome also appears to change with age [20] (Figure 1). Microbial colonisation in the early stages of life is controlled by environmental exposures, feeding habits, and delivery mode [21]. Infants’ microbiota are predominantly made up of *Lactobacilli* and *Bifidobacteria*, which are important for immunological development and nutrition metabolism [22]. Microbial diversity rises as children grow and then levels off by early adulthood [23], though it declines again with further age [24]. Metabolic activity, and vulnerability to disease, have been linked to these changes [24].

Environmental factors also have an impact, with diets high in fruits, vegetables, and whole grains supporting microbial diversity and linked to a lower incidence of metabolic disease, while diets heavy in fat and sugar can cause inflammation, dysbiosis, and metabolic disorders [25,26,27] (Figure 1). Exercise has been linked to changes in the microbial makeup by encouraging the growth of advantageous bacteria and lowering systemic inflammation [28]. Additionally, pharmaceutical use, especially antibiotics, can have significant consequences on the microbiome by altering it and raising vulnerability to infections and chronic diseases [29].

Furthermore, loss of microbial diversity and possible health consequences have resulted from the homogenisation of microbial communities across geographic regions, brought about by urbanisation and globalisation [23].

### 2.2. Functions

Fermentation, which releases energy from food, is facilitated by the microbiome. Bacteria, fungi, and other microorganisms collaborate in the stomach to break down fibres, complex carbohydrates, and other indigestible substances [30,31] (Table 2).

These food substrates are metabolised during fermentation to produce a variety of by-products, most notably SCFAs like butyrate, propionate, and acetate, which are subsequently used as energy sources [31].

The microbiome is essential for regulating the bioavailability of micronutrients through many processes, such as influencing absorption rates and enzymatic activity [32]. Phytases are enzymes that help release inorganic minerals in bioavailable forms from food, and SCFAs improve mineral solubilisation and transepithelial transport [31]. There is also a relationship between the percentage of the gut microbiome that produces SCFA and the rate of calcium absorption, indicating a correlation between microbial metabolism and mineral status [33].

However, the food consumed and the makeup and variety of the microbiota can affect how efficiently energy is extracted. Alterations in fermentation patterns and energy metabolism can result from dysbiosis, or an imbalance in the composition of the gut microbiota, potentially contributing to metabolic illnesses including obesity and diabetes [34].

*Bifidobacterium*, *Bacteroides*, and *Enterococcus* are known to, partly through dietary precursor conversion, synthesize B and K vitamins, which are useful for various processes, including the clotting cascade [35,36]. However, while this production ability is important, their optimal use depends on production location. Although the colon synthesizes vitamin B12, the ileum is the only place where it is absorbed, restricting the impact of colonic microbes on vitamin B12’s bioavailability [37,38].

Through modification of transporter expression, gut bacteria may also control the kinetics of vitamin C absorption [39], while supplementation of vitamin C has demonstrated increased microbial diversity, encouraging growth of beneficial bacteria and improving synthesis of SCFA [40].

Training the immune system—through interactions with gut-associated lymphoid tissues (GALTs) found on the mucosal lining, which act as a physical–chemical barrier, as well as epithelial cells [41,42]—is also an important role.

The epithelial cells lining the gastrointestinal tract are conduits between bacteria and the immune system. Due to pattern recognition receptors (PRRs), such as Toll-like receptors (TLRs) and nucleotide-binding oligomerisation domain (NOD)-like receptors, they can recognise pathogens and elicit immune responses. When substances generated from gut bacteria activate epithelial cells, they create a variety of cytokines, chemokines, and antimicrobial peptides, influencing the local immune response and aiding in the preservation of immunological homeostasis [41].

GALTs are essential components of mucosal lymphoid tissues, which are continually in contact with the external environment and may serve as the main entrance point for pathogens [41]. GALTs contain many myeloid and lymphoid cells, which shield the host from potentially harmful microorganisms and tolerate commensal microbiota and food-derived antigens [41,43].

## 3. Alterations in the Gut Microbiome in IBD

### 3.1. Dysbiosis

**Figure 2:**
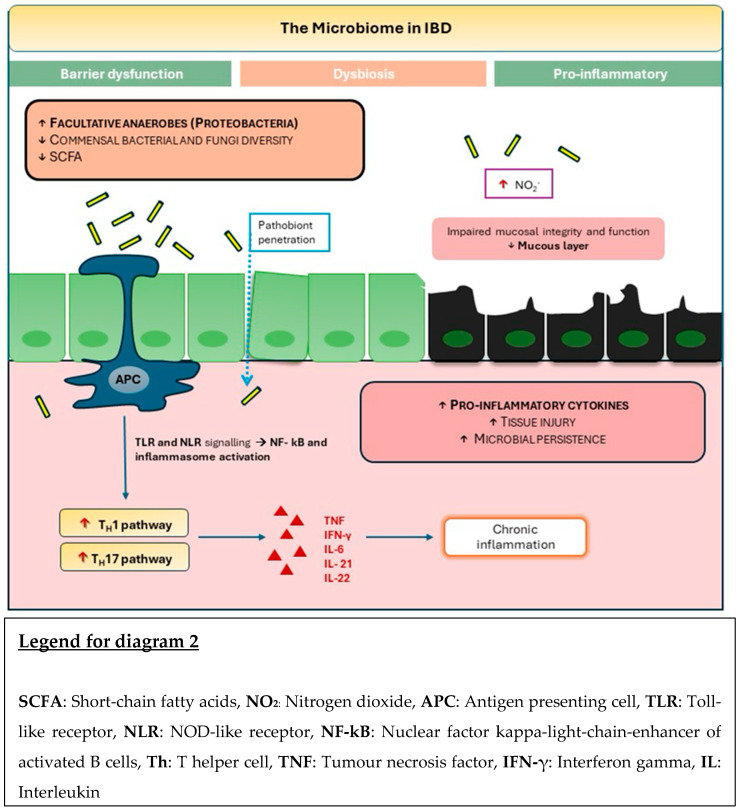
The Microbiome in IBD.

Dysbiosis associated with IBD has been consistently shown to produce fundamental changes to the composition and diversity of the gut microbiota, with a clear reduction in the diversity of commensal bacteria. Other key composition shifts include an increase in *Proteobacteria* and *Enterobacteriaceae* bacterial species. Alongside a decrease in the commensal *Firmicutes*, there is a significant decrease in other favourable bacterial genera, including *Lactobacillus*, and *Eubacterium* [7] (Figure 2).

Other studies further elucidate gut microbial changes in IBD, identifying a reduction of *Christensenellaceae*, *Coriobacteriaceae*, and *Clostridium leptum* in patients with CD, whilst *Actinomyces*, *Veillonella*, and *Escherichia coli* species are in greater abundance [44]. Further studies report increased quantities of pro-inflammatory *Ruminococcus gnavus* in an IBD patient’s microbiome, yet reductions in *Bifidobacterium adolescentis*, *Dialister invisus*, *Faecalibacterium prausnitzii*, and *Clostridium cluster XIVa* [45].

Attempts to identify a preclinical gut microbiome signature that increases the risk of developing CD have found that an increase of the genus *Faecalibacterium* was inversely associated with the risk of developing CD [46]. A reduction in *F. prausnitzii* has been found in many patients with CD [8] and seems to be predictive of more severe disease, reflected by the Crohn’s Disease Activity Index, C-reactive protein levels, erythrocyte sedimentation rate, and serum albumin levels [47]. Other studies have shown that loss of species diversity is also correlated with the disease activity index of CD patients.

*F prausnitzii* and its protective role in CD has been linked to its anti-inflammatory effects, with the supernatant of cultures of *F. prausnitzii* found to be rich with anti-inflammatory factors. This includes a 15 kDa protein found in animal models to prevent the inflammatory signalling pathway NF-κB in intestinal epithelial cells, thereby preventing gut inflammation [48].

### 3.2. Functional Changes

Our gut microbiota may be the nexus between health and disease; dysbiosis leads to perturbations in gut metabolites and micro flora-associated functions. Every bacterial species has a unique mechanism by which it contributes to gut inflammation, including interference with immune-mediated pathways, notably Th17/Treg cells, and the disequilibrium of fermentation products, including SCFAs, carbohydrates, and vitamins [49].

The depletion of *Faecalibacterium prausnitzii*, *Roseburia intestinalis*, *Eubacterium hallii*, *Gemmiger formicilis*, *Eubacterium rectale*, and *Ruminococcus bromii* in patients with IBD leads to a reduction in butyrate. In addition, SCFAs display immune-modulatory activity, including Treg cell development, cytokine production, and anti-inflammatory effects [50].

Intestinal mineral metabolism is disturbed through a decrease in *Collinsella aerofaciens*, *Ruminococcus torque*, and *Bifidobacterium longum.* These bacteria play a role in iron metabolism, bile acid metabolism, and urea cycle metabolism, respectively. Ning et al. also found an enrichment of organic acids related to the tricarboxylic acid cycle in IBD patients, reflecting the aberrant energy metabolism of intestinal microbiota [49].

Conversely, isolate strains of *Ruminococcus gnavus* have robust pro-inflammatory activity through the production of polysaccharides and mucin-degrading trans-sialidase that promote inflammation by compromising the intestinal mucosal barrier [51]. Several studies have shown that *Candida albicans* is abundant in patients with IBD; certain high-damage strains exacerbate intestinal inflammation by inducing a Th17 cell inflammatory response [52].

### 3.3. Microbial Interactions

Studies have linked IBD with biofilm formation and postulate its pro-inflammatory role. Biofilms are complex ecosystems of microorganisms embedded in self-produced matrices of extracellular polymeric substances (EPSs) that adhere to biological surfaces [53].

Biofilms perpetuate immune-mediated inflammation in CD by direct stimulation of epithelial and immune cells; they penetrate the gut epithelium’s mucus layer and adhere to the epithelial interface [54]. Colonic epithelium in CD has been shown to harbour biofilms dominated by *Bacteriodes fragilis*, *Enterobacteriaceae*, *Helicobacter pylori*, *Pseudomonas aeruginosa*, and *E. coli* [55].

The pathobiont adherent-invasive *E. coli* (AIEC) strain LF82 forms intracellular bacterial communities with biofilm-like traits [56]. The biofilms produced by AEIC express components often present within Enterobacteriaceae biofilms; these include curli, cellulose, type 1 pili, flagellin, and extracellular DNA. These components have been shown to impact colitis activity by upregulating mucosal immune responses and modulating epithelial barrier function via TLRs, NOD-like receptors, and carcinoembryonic antigen-related cell adhesion molecules [57].

Bacteria use quorum sensing to interact and communicate with each other. Quorum-sensing molecules, notably n-acyl homoserine lactones (AHL), are secreted by bacteria, and these affect gene expression and drive biofilm formation and the expression of virulence factors. Interestingly, AHL levels may correlate to the intestinal inflammatory state and therefore may provide a useful marker of disease activity in CD [58].

## 4. Factors Affecting the Middle Eastern Microbiome

### 4.1. Diet and Nutrition

The Middle Eastern diet has undergone drastic change, from traditional to industrial [59]. Previously replete with fibre-rich foods such as legumes and fermented foods like yogurt and cheese, it has transitioned to a more western-style diet, with high levels of fat, sugar, and sodium [60].

Fibres come in two types: soluble and insoluble. They affect the microbiome differently, with fruit- and vegetable-derived fibres increasing numbers of gram-positive bacteria such Clostridia, while fibres from legumes increase the number of Bifidobacteria [61].

Fermented foods also affect microbiome composition [62], with the consumption of fermented plant-based foods linked to bacteria associated with fermented foods, like *Lactobacillus* [63].

Diet, environment, genetic factors, and microbiome all play a role in the development of IBD [64]. The incidence and prevalence of IBD is rising in the Middle East and has been partly attributed to the adoption of more western diets [1,65]. Although the prevalence of IBD in the Middle East is relatively low, there is an increasing trend [2], with increases in Kuwait [66], Saudi Arabia [67], Bahrain [68], Oman [69], and Lebanon [70].

### 4.2. Genetics

With genome-wide association studies, various disease susceptibility loci markers for IBD—mostly high-frequency allelic variants—have been identified [71]. These include missense variants found within coding regions of genes, such as Nucleotide-binding Oligomerisation Domain-containing 2 (NOD2), Autophagy-related 16-like 1 (ATG16L1), and Interleukin 23 Receptor (IL23R) [72]. Three single nucleotide polymorphisms (SNPs) have been identified as having individual associations with CD in particular: rs2066844, rs2066845, and an insertion mutation known as 3020insC [73].

Most IBD genome-wide association studies (GWAS) have been conducted in Caucasian or Asian populations, leaving a gap in understanding population-specific variations in the Middle East [74]. When NOD2 (P268S and R702W) and IL23R (G149R and R381Q) variations, identified from GWAS in Caucasian populations, were assessed as potential genetic indicators for IBD in Saudi patients [9], it was found that of the four SNPs, IL23 [rs76418789, rs11209026] and NOD2 [rs2066842, rs2066844], three [rs2066844, rs76418789, rs11209026] were uncommon among IBD patients and controls in the Arabian population. The rs2066842 variation minor allele frequency (MAF) was higher in the Saudi population than globally, but there was no significant difference in MAF between those with and without IBD. There seemed to be no significant association between the genetic risk markers NOD2 and IL23R in Saudi IBD patients.

In a Kuwaiti study assessing the NOD2 gene variants P268S, IVS8 + 158, G908R, L1007fs, and R702W, however [74], polymorphisms in NOD2/CARD15 were found to be significantly linked to a higher risk of developing CD, as well as more severe forms.

### 4.3. Environmental Factors

Animal studies have shown that exposure to environmental heat stress leads to reduced diversity and lower levels of beneficial bacteria like *Lactobacillus* and *Bifidobacterium* in chickens [75]. Humidity mixed with high temperatures has also been shown to cause microbiota dysbiosis, with increased numbers of *Firmicutes*, *Candidatus Saccharimonas* and *Lachnoclostridium* [76].

Urbanisation has been implicated as one of the environmental factors leading to an increase in the prevalence of IBD [77]. This, alongside westernisation of the diet, pollution, use of antibiotics, and changes in hygiene practices, affects gut bacteria and the potential development of IBD [77]. The improvement of hygiene practices has potentially led to an increase in the incidence of IBD, partly due to improved freshwater availability, hot water sources, and smaller families, leading to reduced crowding and uncontaminated food [78]. The increasing use of antibiotics also alters the microbiome, creating an imbalance that can increase the risk of IBD [79].

Research focused specifically on the Middle East is insufficient, however, and more is required to better understand the effects of environmental factors on the prevalence of IBD in the region.

## 5. Therapeutic Interventions

In the Middle East, with IBD prevalence increasing [2], dietary interventions, such as pro- and prebiotics, nutritional modifications, and faecal microbiota transplantation (FMT), offer potential management strategies [80,81].

### 5.1. Probiotics and Prebiotics

Probiotics are microorganisms that can confer a health benefit and are used as adjunctive therapy for IBD [82,83]. The strongest evidence is in pouchitis [84]. RCTs testing the probiotic cocktail VSL3# found a reduction in pouchitis incidence in the 12 months following surgery, as well as reduced rates of refractory pouchitis [85,86]. Probiotics have also shown a trend towards clinical benefit in inducing and maintaining remission in mild–moderate UC, though the results were more mixed than in the studies on pouchitis [87] in both adult [88] and paediatric [89] populations.

The evidence for probiotic use in CD is, however, more limited, with no benefit in patients with active CD, and no prevention of relapse or postoperative disease [90,91,92].

Prebiotics are non-digestible fibres that selectively stimulate the growth and activity of beneficial endogenous colonic bacteria [93]. When administered with probiotics, they are known as synbiotics. Most prebiotics are carbohydrates that are fermented by beneficial colonic bacteria and aid in their metabolism [93]. Prebiotics can have beneficial effects through modulation of the gut microbiome, increasing the number of beneficial bacteria and enhancing their anti-inflammatory effects, such as decreasing the production of pro-inflammatory cytokines or providing nutrition for colonocytes, hence enhancing the repair of injured gut epithelium [93,94]. However, evidence for these beneficial effects is derived largely from animal studies and focused more on irritable bowel syndrome (IBS) than IBD [95].

### 5.2. Diet Modification

A shift from a traditional diet to a westernised one in the Middle East, with increased ultra-processed foods with high contents of fat, sugars, and salt [60], can lead to increased pro-inflammatory cytokine production, altered intestinal permeability, and disruption of the microbiota, fostering a low-level chronic intestinal inflammation, which increases the risk of developing IBD [96,97]. Dietary modifications could therefore play a therapeutic role in IBD.

One example is omega-3 fatty acids, found in fish oils, which decrease the production of inflammatory eicosanoids, cytokines, and reactive oxygen species [98]. Fish consumption has been shown to have an inverse relationship with the development of CD, while increased omega-3 fatty acids intake reduces the risk of the development of UC [99]. Bluefish, a rich source of these fatty acids, has been recommended for IBD patients [96]. However, there is not enough evidence to provide recommendations on the effects of omega-3 fatty acids in active IBD [100].

Antioxidant supplementation for IBD may also be beneficial via its relieving effects on the oxidative/nitrosative stress that plays a key pathophysiological role in IBD [100], including the antioxidant curcumin, which led to significant improvements in clinical and endoscopic parameters, with minimal side-effects [101,102]. Micronutrients with strong antioxidant properties, such as vitamins C and E, have also demonstrated theoretical benefits for IBD, both in animal studies showing reduced inflammation in UC with ascorbic acid [103] and for patients with mild–moderate UC, with a supplement containing antioxidants such as vitamin C, vitamin E, and selenium resulting in a decreased requirement for corticosteroids [104].

However, IBD is a heterogenous condition. As such, any dietary advice should ideally be personalised [105], particularly when considering the microbiome’s response, which varies between individuals [106]. This is true in IBS, where the baseline microbiome composition can predict treatment success [107]. Given that dietary treatments alter the microbiome in CD [108,109], patients’ microbiome compositions could play a role in approaches to personalised dietary recommendations in future [110].

### 5.3. Faecal Microbiota Transplantation (FMT)

FMT involves faecal bacteria from a healthy donor being transplanted into a patient’s GI tract [111]. It is established as a treatment for recurrent *Clostridium difficile* infection (CDI) [112,113] and is gaining interest for IBD [111].

FMT as a treatment for IBD was first documented in 1989, with an FMT enema leading to a patient becoming asymptomatic after six months [114] and remaining in endoscopic and histological remission 20 years later. Since then, several studies conducted have shown potential benefit in both adult [111,115] and paediatric [116] populations.

Despite widespread evidence indicating FMT’s safety, however [117,118], concerns have been raised about adverse effects, with 2.9% of patients with IBD flares who were treated with FMT for CDI subsequently hospitalised with IBD flares [119].

Implementing FMT in the Middle East also comes with challenges. There is a paucity of FMT research in Middle Eastern countries, with none of the 384 studies on FMT between 2010 and 2019 involving Middle Eastern countries [120]. This may feed into a lack of awareness around FMT there, with Iranian [121] and Qatari [122] healthcare professionals (HCP) being unfamiliar with FMT and the role of gut microbes in patient health.

There are cultural and religious barriers, as well, with 30% of Jordanian HCPs expressing a need to consider religious points of view regarding FMT’s permissibility [123] and the consideration of dietary restrictions regarding alcohol or non-halal foods, which may cause consent issues. Additional screening of donors’ dietary habits may be needed to alleviate this [123].

## 6. Future Directions

### 6.1. Precision Medicine

IBD is influenced by many factors, contributing to variability in disease presentation and progression. Precision medicine holds promise in IBD management, integrating factors including genetics, epigenetics, gene expression, and metabolites. Precision medicine is an evolving field that has the potential to advance early detection, optimise resource allocation, and improve outcomes [124]. Our understanding of the underlying processes of IBD, which arises from multiple mutations [125], is advancing, with a focus on the roles of the genome, exposome, microbiome, and immunome, allowing us to potentially practice precision medicine within a multiomics approach [126].

The effectiveness of certain treatments, including FMT, probiotics, and prebiotics, is influenced by a patient’s microbiota [127], with an optimal approach, therefore, considering specific microbiome profiles before starting or changing therapy. Indeed, a randomised controlled trial is currently investigating the effects of personalised microbiota-based therapy, involving antibiotics and prebiotics in pouchitis (NCT04082559).

### 6.2. Microbiome Modulation

Bacteriophages are small viruses known for their ability to infect and replicate within bacteria. They employ various methods to penetrate the bacterial cell wall, subsequently releasing genetic material, allowing the generation of new phage particles, which can go on to infect neighbouring bacterial cells [128]. This has shown promise in providing protection against intestinal injury and infections, potentially allowing bacteriophages to target specific bacteria without harming beneficial resident bacteria [129].

With developments in genetic modifications (GM), GM bacterial or probiotic strains have been created that serve as “intestinal biosensors”, detecting inflammatory markers, or as “resident cell factories”, producing therapeutic molecules that enhance drug delivery at the mucosal surface [124,130]. The primary focus has been on introducing plasmids encoding immunoregulatory cytokines, reporter substrates, or anti-inflammatory mediators into bacterial cells. Plasmids are small circular DNA molecules that exist independently of chromosomal DNA and can replicate autonomously. Their ease of transfection into bacterial cells has made them a prominent vector for introducing recombinant DNA expression into specific probiotic strains. A typical plasmid comprises essential DNA sequences, including a DNA replication origin—an antibiotic resistance gene, used to select transfected bacteria from non-transfected ones—and the “Multiple Cloning Site”, where exogenous DNA fragments are inserted using restriction enzymes. Through recombinant DNA techniques, desired genes can be inserted into a plasmid, which may also include promoter regions to control plasmid expression chemically [130].

*E. coli* strains, especially *E. coli Nissile 1917* (EcN), are used widely for genetic bioengineering. The popularity of EcN can be attributed to the stability and non-transferability of its two small cryptic plasmids, pMUT1 and pMUT2 [130]. Additionally, the chromosome of EcN exhibits high genetic stability, with an absence of sequence changes after 100 consecutive passages in vitro and the intestinal tract [131]. Furthermore, incorporating EcN into bioengineering strategies can combine its immunomodulatory effects on the mucosal immune system with its capacity to produce and release anti-inflammatory molecules [124]. Animal studies showed that EcN bacteria, genetically modified to produce the immunoregulatory protein Sj16 from the helminth *Schistosoma japonicum*, improved colitis in mice by influencing the microbiota composition [132]. The bacterial Sj16 peptide demonstrated protective effects against colitis by targeting peroxisome proliferator-activated receptor alpha (PPAR-α) receptors and restoring the population of the *Ruminococcaceae* family, increasing butyrate levels in the intestine [132]. Modifying the metabolic pathway of EcN to enable direct production of (R)-3-hydroxybutyrate (3HB) may also help, as 3HB modulates macrophages to reduce the production of interleukins and restores the balance between Treg and Th17 [133]. Additionally, the in situ release of anti-inflammatory interleukins in a mouse model of colitis, via *E. coli* engineered to produce IL-35, successfully reduced the inflammatory response by downregulating Th17 cells [134].

Phage therapy has the potential to complement antibiotics, though its appeal for investment is limited by difficulties in patenting the technology [134], as well as consent issues due to uncertainty surrounding risks. Phages also target specific bacteria, unlike antibiotics, making treatment failure potentially more likely if the diagnosis or mechanism of delivery is wrong [135].

### 6.3. Population Studies

Collaborative efforts among Middle Eastern countries to conduct large-scale microbiome studies is needed for developing customised interventions and guiding public health policy. By combining resources and expertise, researchers can gather comprehensive data on microbiome profiles, allowing the identification of patterns, unique variations, and potential associations between microbiota composition and health conditions in the Middle East, potentially facilitating the development of region-specific probiotics or microbiome-based therapies.

## 7. Conclusions

IBD is a complex condition, with many underlying factors contributing to its development and severity, including genetics and microbiome composition. While we know increasing amounts about the bacteria that make up the microbiome, as well as the roles they play, such as *Firmicutes* and the conversion of fibre into butyrate or *Bacteroidetes* and their role in vitamin synthesis, more needs to be discovered regarding how they influence the risk of developing certain conditions.

In the IBD context, there does seem to be a link between risk of development of Crohn’s disease and certain bacterium such as *Faecalibacterium* or *F. prausnitzii*. Biofilm formation may also play a role in the pro-inflammatory states found in IBD.

Changes in the traditional Middle Eastern diet, along with subsequent effects on the microbiome, have also seemingly contributed to increased rates of IBD in the region. Meanwhile, though genetic analyses have identified multiple susceptibility loci markers in mostly Caucasian populations, the few studies on these in the Middle East populations have indicated that this may not apply there.

As we hone this information further, personalised therapies, with probiotics, dietary modification, FMT, or even genetically modified phage therapy, become more of a possibility.

Across the Middle East, going forwards, consistency in study design, sampling protocols, and sequencing techniques is essential to ensure reliability of results, allowing the comparison of microbiome profiles and identifying true differences and associations across populations.

## Figures and Tables

**Table 1:** Keywords used in literature search of databases.

‘Inflammatory bowel disease’, ‘IBD’, ‘Ulcerative Colitis’, ‘UC’, ‘Crohn’s Disease’, ‘CD’, ‘Microbiome’, ‘Firmucutes’, ‘Bacteroidetes’, ‘Dysbiosis’, ‘Middle East’, ‘Arab’, ‘Diet’, ‘NOD’, ‘Probiotics’, ‘Prebiotics’, ‘Faecal Microbiota Transplantation’,

**Table 2:** Functions of the Microbiome.

Phylum	Role in Gut Microbiome	Genera
*Firmicutes*	Fermentation: Degradation of dietary fibres into short-chain fatty acids (SCFAs) such as butyrate, an energy source for colonocytes. Gut Barrier: Supports the integrity of the intestinal barrier. Immune Regulation: Mediates inflammatory reactions and the immune response, such as colonic Treg cells.	*Lactobacillus* *Clostridium* *Enterococcus*
*Bacteroidetes*	Carbohydrate Digestion: Produces SCFAs such as acetate and propionate by breaking down complex carbohydrates. Metabolic Regulation: Expresses bile salt hydrolases, important in bile acid metabolism. Immune Regulation: Mediates inflammatory reactions and the immune response, such as promotion of CD4+ T cell differentiation.	*Bacteroides* *Prevotella*
*Actinobacteria*	Metabolic Regulation: Produces acetate, a co-substrate for butyrate, an energy source for colonocytes. Immune Regulation: Maintains gut barrier homeostasis and induces colonic Treg cells.	*Bifidobacterium* *Corynebacterium*
*Proteobacteria*	Pathogenicity: When overgrown, associated with dysbiosis and intestinal inflammation, increasing risk of metabolic syndrome and IBD. Metabolism: Fixation of nitrogen.	*Escherichia* *Salmonella* *Helicobacter*
*Verrucomicrobia*	Mucin Degradation: Helps to break down mucin, producing metabolites such as SCFAs. Immune Regulation: Produces antimicrobial peptides.	*Akkermansia*
*Fusobacteria*	Amino Acid Metabolism: Contributes to the metabolism of peptides and amino acids. Pathogenicity: Associated with dysbiosis and inflammation in the gastrointestinal tract, as well as colorectal cancer.	*Fusobacterium*

## Data Availability

No new data were created or analysed during this study. Data sharing is not applicable to this article.
